# Sustaining Local Production of Influenza Vaccines: A Global Study of Enabling Factors Among Vaccine Manufacturers

**DOI:** 10.3390/vaccines13111160

**Published:** 2025-11-14

**Authors:** Christopher Chadwick, Claudia Nannei, Erin Sparrow, William Ampofo, Antoine Flahault, Seth Berkley

**Affiliations:** 1 Institute of Global Health, Faculty of Medicine, University of Geneva, 1211 Geneva, Switzerland; 2 World Health Organization, 1211 Geneva, Switzerlandsparrowe@who.int (E.S.); 3 Department of Medical Laboratory Sciences, School of Biomedical and Allied Health Sciences & Virology Department, Noguchi Memorial Institute for Medical Research, College of Health Sciences, University of Ghana, Accra 23321, Ghana; 4 Pandemic Center, School of Public Health, Brown University, Providence, RI 02912, USA

**Keywords:** influenza vaccine, local production, access to vaccines, epidemic and pandemic preparedness, sustainable production

## Abstract

**Background/Objectives:** Local production is a global priority for increasing access to routine, outbreak, and pandemic vaccines and leads to a variety of direct and indirect benefits for countries. This study aimed to characterize the enabling environment for the sustainable production of influenza vaccines, including for epidemic and pandemic preparedness. **Methods:** National/local vaccine manufacturers were surveyed to capture data on influenza vaccine market contributions, government support for local production, and involvement in national pandemic preparedness activities. Using a conceptual framework for sustainable local production of influenza vaccines for epidemic and pandemic preparedness, manufacturers described 41 global/regional, national, and institutional sustainability factors across policy, health system, research and development (R&D), and regulatory thematic domains. In addition to the survey, key findings from country-level sustainability assessments of vaccine production in Bangladesh, Brazil, Indonesia, Serbia, and Viet Nam were analyzed to complement survey results. **Results:** This study included 12 participants representing 11 manufacturers from 10 countries. Of the 11 manufacturers, six reported that their countries have policies that support local production, but most manufacturers reported benefiting from some level of direct or indirect support by the government. Manufacturers considered 40/41 factors as important for sustainable production of influenza vaccines, and among the four domains, influenza prevention and control policies, influenza burden data, quality management, and regulatory filing capacity ranked highly. Additionally, manufacturers ranked factors related to cohesive policies for local production promotion and business/strategic planning at the manufacturer level as the top sustainability factors. **Conclusions:** Manufacturers broadly agreed on the importance of cohesive policies, evidence-based public health priorities, robust R&D and manufacturing investments, and regulatory readiness, though perceptions varied across contexts and company characteristics. Sustainable local production of influenza vaccines should be driven by the alignment of policies, investments, and demand.

## 1. Introduction

Global stakeholders recognize that a critical component for achieving universal health coverage is improved access to quality, safe, effective, and affordable medicines and vaccines. However, the inequitable and inadequate supply of medicines and vaccines, high prices, and out-of-pocket payments drive inequities among vulnerable populations. Improving access to medicines and vaccines relies on affordability, availability, and appropriateness, especially if the medicines and vaccines are locally adapted. Countries have identified local production of vaccines (i.e., production by national manufacturers serving a portion of the domestic market or developing country vaccine manufacturers as opposed to multinational companies (MNCs)) as a high-level political and strategic priority as it can support increased access to vaccines in low- and middle-income countries (LMICs) through increased competition, increased economies of scale, and diversified geographic access. Additionally, local production can strengthen national security and enhance industrial and economic development; however, vaccine prices will be higher at least initially and possibly in the long-term as well, requiring sustained government willingness to pay premiums to realize health, national security, industrial, and economic benefits [1,2].

Among the direct benefits of local production of vaccines are supply reliability and foreign import savings; indirect benefits include the development of innovation capacity, export capacity and experience, and human capital. However, barriers to local production can be substantial—especially for new local manufacturers—and include challenges with securing initial investment, high operating costs, long project durations, limited partnership opportunities, and availability of a highly skilled workforce [1,3]. Governments can support local production directly (e.g., through grants, subsidies, tax and duty exemptions for imported inputs for local production, advanced market commitments, and other incentives) and indirectly (e.g., through facilitating access to foreign markets and development of pooled procurement mechanisms; developing appropriate pricing policies, appropriate intellectual property (IP) regimes, and investment policies) [4].

Inequitable access to vaccines during epidemics and pandemics continues to be a challenge and threatens global, regional, and national health security. Insufficient production capacities, limited to no advance purchase agreements with LMICs, and delays in the deployment of vaccines are just a few of the drivers of inequitable access. In 2006, the World Health Organization (WHO) developed the Global Action Plan for Influenza Vaccines (GAP) to address shortages in influenza vaccines in the event of an influenza pandemic, including by establishing production capacity in LMICs through technology transfer and promoting uptake of seasonal influenza vaccines to sustain pandemic influenza vaccine production capacity [5]. For the 2009–10 influenza A(H1N1) pandemic, global production capacity was insufficient or nonexistent in several regions and largely concentrated in high-income countries (HICs), and the deployment of pandemic influenza vaccines to eligible countries began in December 2009 when donated vaccines became available, almost six months after the pandemic declaration [6,7]. Building upon GAP’s objectives, seasonal influenza vaccination programs have been hypothesized to strengthen pandemic vaccine responses, including for the influenza A(H1N1) and COVID-19 pandemics [8,9]. In 2022, WHO updated its influenza vaccination position paper to recommend that countries consider establishing influenza vaccination programs to both protect vulnerable populations and strengthen capacity for pandemic influenza preparedness and response [10].

Given the vaccine supply inequities experienced during the COVID-19 pandemic response, global calls for local and regional production of vaccines have intensified, which led to the establishment of various political and programmatic initiatives, including G7 and G20 political support, the mRNA Technology Transfer Programme, the Partnerships for African Vaccine Manufacturing, and the African Vaccine Manufacturing Accelerator [11,12]. Additionally, the Regionalized Vaccine Manufacturing Collaborative was launched in 2022 by the World Economic Forum, the U.S. National Academy of Medicine, and the Coalition of Epidemic Preparedness Innovations (CEPI) to advance vaccine equity and strengthen health security for all regions by establishing regional vaccine manufacturing and supply chain networks. Now in its second phase with CEPI hosting the Secretariat, the Regionalized Vaccine Manufacturing Collaborative is actively collaborating with key regions to enhance vaccine production capabilities in partnership with regional bodies, governments, industry, funders, and other critical stakeholders [13].

Most recently, in May 2025, the Seventy-eighth World Health Assembly adopted the Pandemic Agreement through resolution 78.1, calling for a variety of measures to achieve sustainable and geographically diversified local production, including skills development, public and private sector investments, advanced purchase agreements, and public-private partnerships [14]. Previous frameworks have addressed the enabling environment for local production of vaccines, recognizing the interplay between supply and demand. One framework was developed under GAP and considered the sustainability of local production of influenza vaccines in terms of six factors: the policy environment and healthcare system, surveillance systems and influenza disease and economic burden, product development and manufacturing, product approval and regulation, communication to support influenza vaccination, and financing [15,16]. Additional frameworks have been developed to address the enabling environment, including the viability of manufacturers and the Health Vaccines Market Framework [17,18,19].

Given the global focus on local production of vaccines for epidemic and pandemic preparedness and the expansion of influenza vaccine production to LMICs, we aimed to better understand what should be considered for sustainable production of influenza vaccines. In this study, we specifically sought to:

• Characterize influenza vaccine production and supply practices among national/local vaccine manufacturers;
• Assess attitudes and perceptions of sustainability factors for local production of influenza vaccines.

## 2. Materials and Methods

### 2.1. Conceptual Framework

Table 1 outlines the enabling environment for the sustainable influenza vaccine production framework utilized for this study. This framework was adapted from previously developed frameworks that addressed sustainable vaccine production from the perspectives of policymakers, public health program managers, manufacturers, and regulators. Through the review of these frameworks, sustainability factors can be organized into the four domains that are described in Table 1.

### 2.2. Study Participants

The inclusion criteria for the study were established national/local vaccine manufacturers that currently produce or previously produced an approved seasonal, pre-pandemic, or pandemic influenza vaccine. MNCs were excluded from the study. Through literature and website reviews, including individual company websites and websites for the International Federation of Pharmaceutical Manufacturers and Associations, Biotechnology Innovation Organization, and Developing Countries Vaccine Manufacturers Network [20,21], 59 influenza vaccine developers and manufacturers, representing MNCs, biotech, and national/local manufacturers, were identified, of which 34 were relevant for the study based on the inclusion and exclusion criteria.

### 2.3. Data Collection

The survey, which is available in the Supplemental Material (Document S1), was designed using the LimeSurvey platform (LimeSurvey GmbH, n.d.) (Version 6) to capture information on the characteristics and capabilities of manufacturers; enabling environment for influenza vaccine production, including influenza vaccine market contributions, government support, and pandemic preparedness; and sustainability factors. Perceptions of the importance of sustainability factors were assessed on a 5-point Likert scale with 1 being “not at all important” and 5 being “critically important.” Online links for the survey were shared with influenza vaccine R&D, manufacturing, or management focal points among manufacturers via email, along with instructions for completing the survey and a fillable Microsoft Word version of the survey to support data collection. Participants were encouraged to use the fillable Microsoft Word version of the survey to facilitate data collection with relevant colleagues prior to completing the online survey. Participants were informed that information collected in the survey would remain confidential and secure, and final data would be anonymized. The survey was open from March to September 2023. Up to three reminders were sent.

To complement survey data, relevant publicly available country-level assessments were identified for Bangladesh, Brazil, Indonesia, Serbia, and Viet Nam through a literature review.

### 2.4. Data Analysis

Survey data were compiled in LimeSurvey and exported to SPSS Statistics (IBM SPSS Statistics for macOS, version 29.0, Armonk, NY, USA) for cleaning and analysis. Focal points identified in the survey were contacted if there were missing data or for clarifying questions. Descriptive statistics were generated for all survey data, including manufacturer characteristics, enabling environment, and sustainability factors.

For additional analyses, the sustainability factors were grouped into the four domains described in Table 1, and ordinal data were analyzed using nonparametric tests suitable for non-normally distributed data. To assess agreement on the level of importance of sustainability factors, the Friedman test was used; Kendall’s coefficient of concordance (W) was used to measure the degree of agreement among manufacturers. Manufacturers were organized into a series of sub-groups to determine if agreement was influenced by certain characteristics or capabilities: (1) company management structure, (2) company size, (3) country classification by income level, (4) influenza vaccine production outputs, (5) public and/or private vaccine market participation, (6) Northern Hemisphere (NH) and/or Southern Hemisphere (SH) influenza season supply, and (7) influenza vaccine export status. Statistical significance for the Friedman test was set at *p* < 0.05. Kendall’s W ranges from 0 (no agreement) to 1 (perfect agreement). When W ≥ 0.5 within sub-groups, agreement is characterized as moderate to strong consensus. To assess differences among perceptions of individual factors between groups, Mann–Whitney U and Kruskal–Wallis H tests were used for comparisons between two groups and among three groups, respectively. Where statistical differences were found with the Kruskal–Wallis H tests, post hoc pairwise comparisons were conducted. Statistical significance was set at *p* < 0.05, except when a Bonferroni adjustment was applied for the Kruskal–Wallis H test, and significance was set at *p* < 0.017 when three pairs were compared.

Country-level assessments were reviewed, and relevant findings based on the survey findings were extracted and organized by the four sustainability domains in Table 1. Key findings from these sustainability assessments were analyzed to identify areas of convergence and divergence with the survey data, providing complementary qualitative insights.

## 3. Results

### 3.1. Participant Characteristics

Twelve individuals from 11 local/national vaccine manufacturers completed the survey, representing a 32% response rate (11/34 manufacturers). These manufacturers are in Argentina, Bangladesh, Brazil, China, India, Indonesia, Republic of Korea, Serbia, Thailand, and Viet Nam. Participants provided details on their company’s management structure, annual installed production capacity for all vaccines, landscape of vaccines that have been approved by an NRA or achieved WHO prequalification (PQ), the presence of procurement agreements with their country’s government, the supply of influenza vaccines to a regional and/or global procurement mechanism, and the presence of other influenza vaccine suppliers in country. Characteristics of the 11 participating manufacturers are described in Table 2.

### 3.2. Enabling Environment

#### 3.2.1. Influenza Vaccine Market

**Figure 1:**
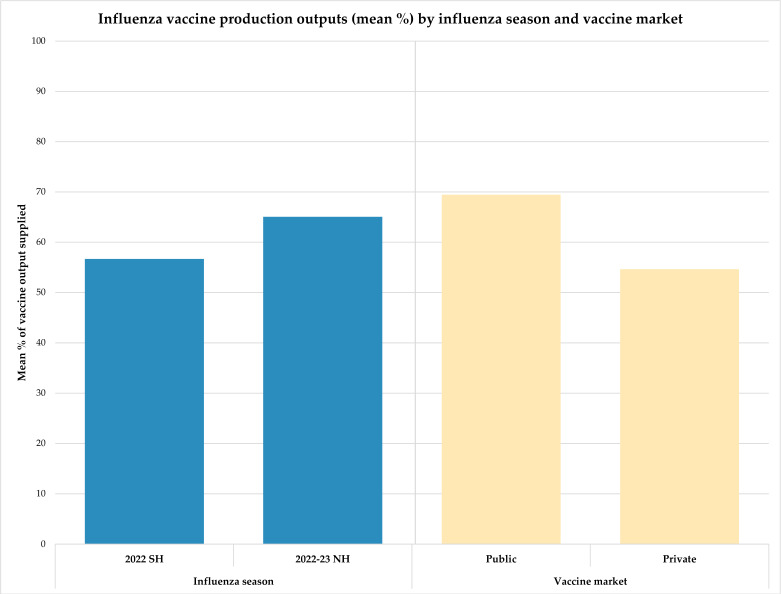
Mean percentage of influenza vaccine production outputs reported by manufacturers (*n* = 10) for the 2022 SH and 2022–23 NH influenza seasons and for the public and private vaccine markets.

Ten of the 11 manufacturers provided data on the percentage of their seasonal influenza vaccine production outputs supplied to the public and/or private markets for the 2022 SH and 2022–23 NH influenza seasons. Actual volumes supplied were considered sensitive and not collected in the survey. Obtaining percentages allowed for comparison of the role the manufacturers have in the national market, irrespective of the actual demand. Of the 10 manufacturers, eight reported supplying 100% of production outputs to public and/or private markets for a given influenza season, and four of these supplied influenza vaccines for both influenza seasons. The two participants that did not report 100% outputs supplied are public sector manufacturers, one from a lower MIC and the other from an upper MIC. Figure 1 shows the mean percentages for all 10 manufacturers broken down by influenza season and vaccine market. These data show relatively similar outputs across seasons and market segments, with slightly higher outputs for the 2022–23 NH season and for the public vaccine market across both seasons.

**Figure 2:**
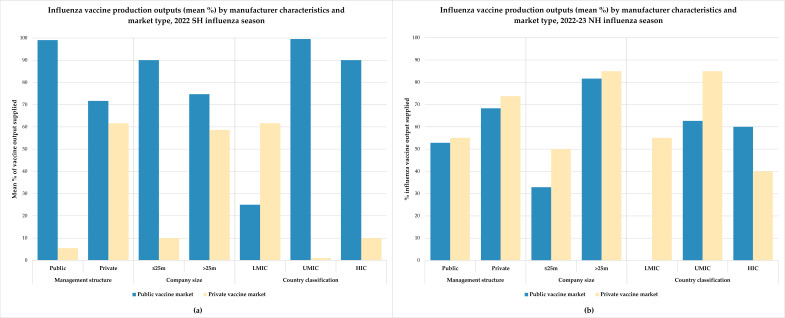
Mean percentage of influenza vaccine production outputs reported by manufacturers for public and private vaccine markets, stratified by company management structure, company size, and country classification by income status for the (**a**) 2022 SH influenza season (*n* = 6) and (**b**) 2022–23 NH influenza season (*n* = 9).

When stratified by company management structure (public sector versus private sector manufacturers), company size (as estimated by annual production capacities of ≤25 million doses or >25 million doses for all vaccines), and country classification by income status, more pronounced differences were observed among the six manufacturers reporting data for the 2022 SH season and nine manufacturers reporting data for the 2022–23 NH season (see Figure 2). Overall, manufacturers reported higher outputs across most categories for the SH season compared to the NH season. However, significant contrasts were seen between the public and private vaccine markets, particularly among the public sector, smaller, and upper MIC-based manufacturers. Interestingly, manufacturers in lower MICs (*n* = 3) appeared to rely more heavily on the private vaccine markets, suggesting limited demand in the public vaccine market, which may be influenced by the absence of national seasonal influenza vaccination policies.

Following the 2009–10 A(H1N1) pandemic, six of the seven manufacturers (86%) that were producing seasonal influenza vaccines at the time, including five from MICs and one from an HIC, observed an increase in seasonal influenza vaccine demand. Most of these manufacturers noted that they responded to the increased demand by increasing their production capacities, while one manufacturer also expanded to global markets. Additionally, during the COVID-19 pandemic, six of the eight manufacturers (75%) that were producing seasonal influenza vaccines at the time, including five from MICs and one from an HIC, observed an increase in seasonal influenza vaccine demand. Only two manufacturers, one from an MIC and one from an HIC, observed increases in seasonal influenza vaccine demand for both time periods.

#### 3.2.2. Government Support

Six of the 11 manufacturers (55%), all of which are from MICs, indicated that their country has policies in place to support local production of vaccines. When asked to describe any relevant policies, participants provided examples of policies and other governmental support, including promotion of the biotech industry and a specific product development program with the Ministry of Health. The manufacturer from Viet Nam indicated that as part of the national vaccination strategy, seasonal influenza vaccines will be included in the national expanded program on immunization in 2030. Additionally, a manufacturer from India highlighted a Gujarat State Biotech Policy 2022–27 that aligns with India’s Production Linked Incentives Scheme 2.0 and aims to make Gujarat a competitive destination for the biotechnology industry, including for vaccine production, through the provision of financial support for capital costs, technology acquisition and upgrade, patent assistance, market development, and employment generation.

**Figure 3:**
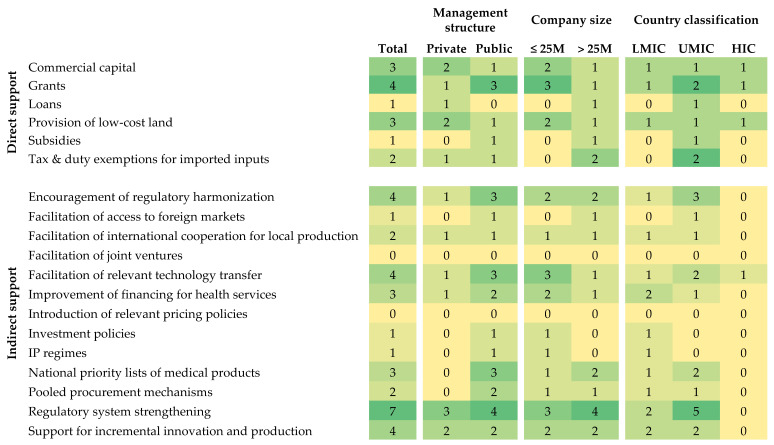
Heatmap of direct and indirect government support for local production, stratified by company management structure, company size, and country classification by income level.

Nine of the 11 manufacturers (82%) indicated some form of direct support by the government for local production of vaccines. Figure 3 provides a heatmap of the direct support observations, which are further stratified by company management structure, company size, and country classification by income status. Overall, four manufacturers benefited from grants, which were reported more often by public manufacturers and smaller manufacturers. Private manufacturers benefited from government support in the form of commercial capital and the provision of low-cost land. Two manufacturers provided feedback on additional forms of direct support, including procurement of equipment and financial support for clinical studies. Ten manufacturers (91%) indicated some form of indirect support by the government for local production, which is described further in Figure 3. Regulatory system strengthening was the most frequently observed form of indirect support, with much of the activity occurring in upper MICs. Other forms of indirect support included encouragement of regulatory harmonization, facilitation of relevant technology transfer, and support for incremental innovation and production. Public manufacturers more frequently reported the benefits of the indirect support compared to private manufacturers.

#### 3.2.3. Pandemic Preparedness

**Figure 4:**
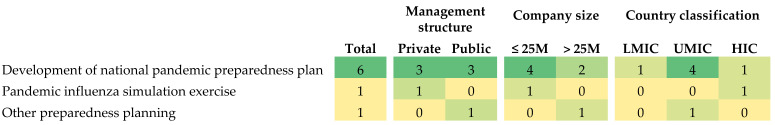
Heatmap of manufacturer involvement in national pandemic influenza preparedness planning, stratified by company management structure, company size, and country classification by income level.

Seven of the manufacturers (64%) indicated involvement in national pandemic influenza preparedness planning. Figure 4 provides a heatmap of the pandemic preparedness planning activities, stratified by company management structure, company size, and country classification by income level. Many of the manufacturers, especially those in upper MICs, have been involved in the development of the national pandemic influenza preparedness plan. However, only one manufacturer indicated participation in a pandemic influenza simulation exercise.

### 3.3. Sustainability Factors

Manufacturers assessed the level of importance of 41 sustainability factors across the four domains: policy coordination and coherence, health system and public health priorities, vaccine R&D and manufacturing, and vaccine approval and regulation. Table 3 provides the median, minimum, and maximum values for the factors. Medians for 40/41 factors were ≥4, suggesting they’re considered important by a majority of participants. Additionally, based upon minimum values ≥ 4, all participants considered 17/41 factors important, with only one factor (capacity to prepare and submit regulatory filings) being considered critically important by all participants, as evidenced by minimum and maximum values of 5.

The Friedman test was conducted to assess the agreement of the importance of sustainability factors within groups, and Kendall’s W was conducted to assess the level of agreement (see Document S2). When looking at all 12 participants from the 11 manufacturers, significant values were obtained for each sustainability domain, suggesting that at least one factor was rated differently. The top factors for each domain included influenza prevention and control policies, influenza burden data, quality management, including current Good Manufacturing Practices (cGMP) compliance, and regulatory filing capacity. However, among all 12 participants, consensus was relatively weak for each domain as Kendall’s W values were less than 0.5. When broken down into sub-groups, differences in the Friedman test and Kendall’s W values were observed. Notable discrepancies from the overall group analyses included the following:

• Policy coherence was rated highly with moderate to strong consensus among larger manufacturers, manufacturers supplying 100% of their outputs, manufacturers supplying a single market (public or private), and manufacturers supplying for a single influenza season.
• Manufacturers that export influenza vaccines had moderate to strong consensus for risk awareness of influenza, seasonal influenza vaccination in the health insurance scheme, and vaccine procurement and delivery strategies/infrastructure.
• Consensus was mostly weak for the R&D and manufacturing domain among sub-groups, but other factors that were rated highly included equipment maintenance, R&D budget and program, demand forecasting and marketing strategy, reinvestment in R&D, and business and strategic plans.
• Public manufacturers and those manufacturers supplying a single vaccine market had moderate to strong consensus for regulatory filing capacity; additional factors that were rated highly included the manufacturer and NRA relationship and NRA maturity level.

Mann–Whitney U and Kruskal–Wallis H tests were conducted to determine if there were any differences in perceived importance among groups, including company management structure, company size, and country classification by income level. To determine if market-related variables had any impact on manufacturers’ perceptions of the importance of the sustainability factors, additional groupings were established, including production output supplied (100% vs. <100%), vaccine market supplied (public and private markets supplied vs. single market supplied), influenza season supplied (NH and SH seasons vs. single season), and influenza vaccine export status (exporter vs. non-exporter). Of the 41 sustainability factors, 19 had at least one significant Mann–Whitney U or Kruskal–Wallis H test (see Document S3). No significant differences were observed for the grouping based on production output supplied. Several significant differences were observed for the country classification and export status groupings. Lower MICs rated policy, health system, and R&D and manufacturing factors higher than HICs, including those related to policy coherence, influenza burden data, influenza vaccine cost-effectiveness data, development/sourcing of raw materials, quality management, and workforce; however, upper MICs rated health system factors higher than HICs, including the national pandemic influenza preparedness plan identifying the local manufacturer and considering seasonal influenza vaccination. Non-exporters rated health system, R&D and manufacturing, and regulatory factors higher than exporters, including local manufacturers participating in pandemic influenza preparedness planning, public awareness of the local manufacturer, business and strategic plans, number of vaccines manufactured, and NRA maturity level.

In addition to assessing the importance of each sustainability factor individually, manufacturers ranked their top 10 factors. Of the 41 factors, 39 were ranked in the top 10 at least once. The other two factors were international sanctions and border control issues, which may have impacts on procuring equipment and influenza viruses for R&D, and access to advocacy networks and partnerships, including industry associations. Sums of the weighted ranks resulted in the following top-ranked factors:

1. Coherence among health, industrial, and economic policies that promote local production;
2. Business and strategic plans to address supply chain issues, market opportunities, and risk mitigation strategies;
3. Demand forecasting and marketing strategy for seasonal influenza vaccines;
4. Quality management, including compliance with cGMP;
5. Budget and program for R&D, process development and optimization, and expansion of capacities;
6. National pandemic influenza preparedness plan takes into consideration seasonal influenza vaccination;
7. Seasonal influenza vaccination included in health insurance schemes or directly provided by the public sector;
8. National pandemic influenza preparedness plan takes into consideration the local vaccine manufacturer;
9. Government/public sector subsidies for local production;
10. Capacity to conduct clinical trials.

As demonstrated by this ranking of factors, manufacturers perceived facility-level and nationally focused health system/public health factors as more important for sustainable production than the policy- and regulatory-level factors.

### 3.4. Country Examples

To further characterize some of the findings from the survey, published assessments for several countries with vaccine manufacturers that participated in the study were identified. Previous assessments of sustainable influenza vaccine production were conducted in Brazil, Indonesia, and Viet Nam [22,23,24], and broader assessments of the ecosystem for local production of vaccines and pharmaceuticals were conducted in Bangladesh and Serbia [25,26]. Notable highlights from these assessments and other resources relevant to this study are outlined in Table 4.

## 4. Discussion

In this study, a cross-sectional analysis of the enabling environment and perceptions of sustainability factors was conducted among 11 national/local vaccine manufacturers. For the 2022 SH and 2022–23 NH influenza seasons, most of the manufacturers supplied 100% of their influenza vaccine outputs to public and/or private markets within their countries. However, there were discrepancies between influenza seasons and vaccine markets among manufacturers when stratified by company management structure, company size, and country income status. Three of the 11 manufacturers supplied influenza vaccines to a global or regional procurement mechanism, such as the PAHO Revolving Fund. While only six manufacturers noted supportive government policies for local production, the majority reported some form of direct and/or indirect support of local production, including grants and regulatory system strengthening, and this support was more common among public manufacturers than private manufacturers.

Twelve representatives from the 11 manufacturers provided their perceptions on sustainability factors by assessing them individually on a Likert scale of 1–5 and holistically by ranking the top 10 factors. Based on median Likert scores, most factors were considered important, which is to be expected since these were identified from previous frameworks looking at sustainable production, viability among national/local manufacturers, and healthy vaccine markets. Interestingly, even though regulatory filing capacity was considered critically important by all participants, it was not ranked in the top 10. There was broad agreement that sustainable local production of influenza vaccines relies on influenza prevention and control policies; coherence among health, economic, and industrial policies; risk awareness of influenza; seasonal influenza vaccination included in health insurance schemes; vaccine procurement and delivery strategies and infrastructure; and regulatory filing capacity. Despite the higher median Likert scores across sustainability factors, differences in perceptions were observed when looking at the four sustainability domains and different sub-groups of manufacturers. When grouped by country income classification, differences in perceptions were observed for several sustainability factors, such that manufacturers in LMICs rated factors related to policy, health systems and public health priorities, R&D and manufacturing, and vaccine regulation and approval higher than HICs. Additionally, when grouped by export status, non-exporters rated several nationally focused factors, such as influenza vaccine cost-effectiveness data, participation in national pandemic preparedness planning, and public awareness of the national/local manufacturer, higher than exporters, which may indicate that the three exporters experience fewer barriers to sustainable influenza vaccine production.

The concept that pandemic influenza vaccine availability and readiness rely on seasonal influenza vaccine production and demand has been hypothesized for decades and was foundational for GAP; it has been further promoted through pandemic influenza preparedness capacity-building supported by the Pandemic Influenza Preparedness Framework [28]. However, upon the sunset of GAP in 2016, it was suggested that the concept had reached its limits due to modest growth in seasonal vaccine demand across regions, and the dogma needed to be challenged [29]. Until there are licensed next-generation influenza vaccines that transform the influenza vaccine market and allow for the de-linkage of pandemic influenza vaccine availability from seasonal influenza vaccine production and demand, our findings suggest that this concept is still important to local/national vaccine manufacturers: the sustainability factor regarding the national pandemic influenza preparedness plan incorporating seasonal influenza vaccination was consistently rated as critically important (median = 5), especially among upper MICs according to the Kruskal–Wallis H tests, and was rated in the top 10 factors. Additionally, for those manufacturers that produced influenza vaccines during the 2009–10 influenza A(H1N1) and COVID-19 pandemics, the majority reported an increase in seasonal influenza vaccine demand. However, just over half reported being involved in pandemic influenza preparedness planning activities in their countries, and none of the manufacturers reported having an advanced purchase agreement in place for pandemic vaccines.

Efforts are underway, especially in Africa, to increase access to vaccine production technologies, including platform technologies such as mRNA and across regions (e.g., Partnerships for African Vaccine Manufacturing and the African Vaccine Manufacturing Accelerator). As access to these flexible platforms increases, the concept mentioned previously may not be as relevant as with current technologies used for influenza vaccines. Additionally, this could be the case if next-generation influenza vaccines that provide broader protection, including for both circulating seasonal influenza viruses and influenza viruses with pandemic potential, become available. However, production capacities will need to be sustained for pandemic preparedness regardless of the vaccines produced in the interpandemic period.

**Figure 5:**
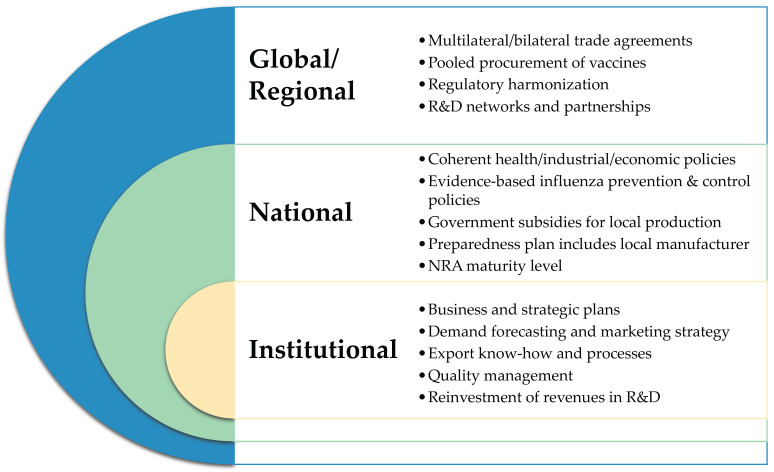
Conceptual model of the enabling environment for sustainable local influenza vaccine production.

Achieving sustainability requires commitment by policymakers, public health program managers, manufacturers, and regulators, as suggested by our findings, where manufacturers emphasized sustainability factors related to policy coherence, business and strategic planning, quality management, and regulatory filing capacity. As illustrated in Figure 5, the sustainability factors identified in this study extend across interconnected global/regional, national, and institutional levels, with stakeholders at each level contributing distinct but complementary roles in enabling access to vaccines, including through local production. Global procurement initiatives and financing mechanisms could be leveraged to stimulate sustainable production capacity by integrating equitable access objectives with health system and industrial investments. Organizations, such as WHO, Gavi, and CEPI, can play critical roles in strengthening this enabling environment, including by supporting regulatory harmonization, facilitating technology transfer and workforce development, and aligning procurement mechanisms to create predictable demand for locally produced vaccines. Regional coordination can optimize resources, harmonize regulatory capacities, provide market access, facilitate pooled procurement, reduce duplication, and ensure benefits are shared across borders as occurs in industries such as aerospace and defense, where multi-country supply chains and shared infrastructure are common [13]. While local production can advance equitable access to vaccines between countries through diversified regional manufacturing, it is important to reemphasize its role in increasing access within countries, especially for those countries where sub-national inequities, including due to vaccine stockouts, remain despite considerable progress made at the national level [30].

The main limitation for this study was the small sample size of participating manufacturers (*n* = 11, 32% response rate), although the number of participating manufacturers represents a considerable number of influenza vaccine manufacturers, as a 2023 survey found 32 established multinational and national manufacturers had bulk manufacturing capabilities, with a few others having fill/finish capabilities [31]. Additionally, although small in absolute terms, the inclusion of 10 current influenza vaccine manufacturers in LMICs is notable as the same survey identified 18 national influenza vaccine manufacturers based in LMICs, underscoring the relevance and representativeness of the findings, especially as the participants represented a diverse group based on four of the six WHO regions, company management structure, company size, and market contributions. Given the inclusion criteria, participants from the WHO African Region were not expected, since there are currently no active influenza vaccine manufacturers in the region. Despite there being some influenza vaccine manufacturers in the WHO Eastern Mediterranean Region, none responded to the invitation to participate in the study. Selection bias may also have influenced the findings, given the study’s focus on manufacturers in MICs currently producing influenza vaccines. Consequently, countries without vaccine production capacity were not represented. This focus may overrepresent contexts with relatively advanced capability and thus limit generalizability to low-income settings. However, the findings still offer valuable insights for those countries, manufacturers, and funders involved in establishing new manufacturing capabilities, particularly since sustainability considerations should be addressed early in the process. Another limitation of the study was its cross-sectional design, and therefore, only a snapshot of market contributions and perceptions of sustainability factors was included in the analysis. Given the use of self-reported survey data from vaccine manufacturers, the possibility of response bias cannot be excluded, especially for Likert-scale data, where respondents may tend to overemphasize the importance of sustainability factors. The study aimed to minimize response bias through the survey design (i.e., use of a 5-point Likert scale with equal positive and negative options) and data collection (i.e., assurance of anonymity and confidentiality to encourage sharing of candid views and perceptions, encouraging review of the survey with colleagues prior to completing it). Finally, perspectives on sustainability were limited to representatives from vaccine manufacturers. However, the use of published case studies allowed for a comparison of study findings with broader findings from countries that were represented in the study.

Future research should incorporate longitudinal analyses of sustainable influenza vaccine production across multiple influenza seasons and years to assess the effects of national policies (e.g., subsidies, procurement schemes, changes in influenza vaccination recommendations), vaccine market fluctuations, and internal manufacturer changes (e.g., new products commercialized). Because sustainability is a multi-stakeholder phenomenon, future research should also incorporate the perceptions of policymakers, public health program managers, and regulators to achieve a more holistic view of what contributes to sustainable vaccine production and how groups prioritize sustainability factors differently.

## 5. Conclusions

This study highlights the complex, multi-domain nature of sustaining local production of influenza vaccines, which relies on more than just production outputs. Sustainability requires coherent policies, evidence-based public health priorities, robust surveillance coupled with R&D strategies, and mature regulatory systems. Our findings show general consensus among a sample of influenza vaccine manufacturers in LMICs for a host of factors that contribute to sustainable production, but perceptions vary across contexts, underscoring the need for tailored approaches and the fact that sustainability is not one-size-fits-all. Additionally, the view that seasonal influenza vaccination supports pandemic preparedness is still a priority among national/local manufacturers but should be reinforced through policy and programmatic initiatives, such as including those manufacturers in pandemic preparedness planning efforts. Country case comparisons further illustrate that sustainability is achievable when policy, investments, and demand align. Achieving sustainable local production of vaccines for epidemic and pandemic preparedness should be viewed as a steady state that needs constant work and commitment from all stakeholders. There is a need to incorporate the lessons from the COVID-19 pandemic for the ever-present health security threat from respiratory diseases, especially influenza. Moving forward, efforts to strengthen pandemic preparedness must also prioritize routine systems and market conditions that support interpandemic vaccine production and access.

## Figures and Tables

**Table 1:** Enabling environment for sustainable influenza vaccine production.

Sustainability Domain	Focus
Policy coordination and coherence	Intersection of health, industrial, and economic policies at the national level and alignment with global and regional norms and standards
Health system and public health priorities	The health system and public health landscape, including surveillance systems and influenza burden evidence, and prevention and control programs
Vaccine research and development (R&D) and manufacturing	Vaccine R&D and manufacturing and manufacturer-level aspects, including facility and business components
Vaccine approval and regulation	The regulatory system, including the national regulatory authority (NRA), in addition to global, regional, and national regulatory aspects for vaccine approvals

**Table 2:** Characteristics of participating manufacturers.

Characteristics	n (%) or Median (Range)
Country classification by income level *	
Low-income	0
Lower-middle	3 (27%)
Upper-middle	7 (64%)
High	1 (9%)
**WHO region**	
Africa	0
Americas	2 (18%)
Eastern Mediterranean	0
Europe	1 (9%)
South East Asia	4 (36%)
Western Pacific	4 (36%)
**Management structure**	
Private	6 (55%)
Public (state/government owned)	5 (45%)
**Annual production capacity for all vaccines (total doses)**	
<5 million	2 (18%)
5–25 million	3 (27%)
26–100 million	3 (27%)
>100 million	3 (27%)
**Product landscape**	
Seasonal influenza vaccines approved by NRA	1 (0–6)
Seasonal influenza vaccines with WHO PQ	0 (0–4)
Pre-/pandemic influenza vaccines approved by NRA	0 (0–4)
Pre-/pandemic influenza vaccines with WHO PQ	0 (0–1)
Non-influenza vaccines approved by NRA	6 (2–90)
Non-influenza vaccines with WHO PQ	0 (0–15)
**Procurement agreements with government**	
Annual/multi-year agreement for seasonal influenza vaccines	3 (27%)
Advanced purchase agreement for pandemic influenza vaccines	0
No agreement	8 (73%)
**Seasonal influenza vaccine supplied to regional/global procurement mechanism**	
Yes	3 (27%)
No	8 (73%)
**Other manufacturer supplies seasonal influenza vaccine in country**	
Yes	10 (91%)
No	1 (9%)

* Based on World Bank classification of country income status (as of 2023).

**Table 3:** Sustainability factors: descriptive statistics for Likert scores (1–5) of importance.

Factor	Median	Minimum	Maximum
**Policy coordination and coherence**			
Coherence among health, industrial, and economic policies that promote local production	5	4	5
Government/public sector subsidies for local production	4	2	5
Impact of multilateral/bilateral trade agreements on commercialization and import/export of products	4	2	5
International sanctions and border control issues	3	2	5
National influenza prevention and control policies, including for vaccination	5	4	5
National policies to generate skilled local biomanufacturing workforce	4	3	5
Political will, stability, long-term vision, and strategic planning	4.5	4	5
**Health system and public health priorities**			
Identification of target groups for influenza vaccination	5	4	5
Influenza disease and economic burden data are known in country	5	3	5
Influenza vaccine cost-effectiveness data are known in country	5	3	5
Local manufacturer is identified as a stakeholder for pandemic influenza preparedness planning	4.5	2	5
National pandemic influenza preparedness plan takes into consideration local vaccine manufacturer	5	2	5
National pandemic influenza preparedness plan takes into consideration seasonal influenza vaccination	5	2	5
Public awareness of local manufacturer/locally produced influenza vaccine	4	1	5
Public awareness of risk of seasonal and pandemic influenza	5	4	5
Regional/pooled procurement of influenza vaccines	4	1	5
Seasonal influenza vaccination included in health insurance schemes or provided by the public sector	5	4	5
Vaccine procurement and delivery strategies and infrastructure	4.5	4	5
**Vaccine R&D and manufacturing**			
Access to advocacy networks and partnerships, including industry associations	4	2	5
Access to R&D networks and partnerships	4	4	5
Budget and program for R&D, process development and optimization, and expansion of capacities	5	4	5
Business/strategic plans to address supply chain issues, market opportunities, and risk mitigation	5	3	5
Chemistry, manufacturing, and controls, including seed development, bulk manufacturing, formulation, yields, and process validation	4	3	5
Competition with multinational manufacturers	4	3	5
Demand forecasting and marketing strategy for seasonal influenza vaccines	5	4	5
Development or sourcing of raw materials (e.g., eggs)	5	2	5
Facility design and construction	4	3	5
Maintenance of equipment	5	2	5
Number of vaccine products manufactured	4	2	5
Procurement, installation, and validation of equipment	4	3	5
Quality management, including compliance with cGMP	5	4	5
Reinvestment of portion of revenues in R&D	4.5	4	5
Strategic selection of technology	4	4	5
Workforce availability, recruitment, retention, and training	5	3	5
**Vaccine approval and regulation**			
Capacity to conduct clinical trials	4	4	5
Capacity to prepare and submit regulatory filings	5	5	5
Export know-how and processes	4	3	5
NRA maturity level	5	4	5
Regional/global regulatory harmonization	5	4	5
Relationship between manufacturer and NRA	5	3	5
WHO PQ	4	3	5

**Table 4:** Country highlights organized by sustainability domain.

Country	Policy Coordination/ Coherence	Health System/ Public Health Priorities	Vaccine R&D/ Manufacturing	Vaccine Approval/ Regulation
Bangladesh	No formal influenza vaccination policy [27] Policies and incentives to support local production of vaccines exist but need to be cohesive	Influenza vaccine provided through the private sector with an average price of USD 9.50; provided free for Hajj pilgrims every year [27] Evidence gaps persist, including for burden among risk groups and cost-effectiveness of vaccination programs	The influenza vaccine manufacturer, Incepta Vaccine Limited, has export experience with some vaccines Academic research networks not well established to support local R&D of vaccines	NRA has not reached maturity level 3 ^a^
Brazil	Influenza vaccination included in the National Immunization Program since 1999 Policies promote national production of vaccines, including financing public production facilities	Priority groups for seasonal influenza vaccination have been identified, and demand is high The influenza vaccine manufacturer, Instituto Butantan, supplies nearly all influenza vaccines in-country, but lack of long-term procurement contracts with Ministry of Health noted as a challenge	Federal initiatives available to train staff but implementation challenges due to budget limitations Production costs considered high, including for supplies, raw materials, and equipment; egg costs are high and account for ~60% of the total cost of the influenza vaccine	NRA is at maturity level 3 ^b^ WHO has prequalified the seasonal influenza vaccine produced by Instituto Butantan (2021) ^c^
Indonesia	No formal seasonal influenza vaccination policy, but the government prioritizes Hajj pilgrims, healthcare workers, and older adults for influenza vaccination Government is committed to local production of vaccines, including through the government-owned manufacturer, Bio Farma, which was established in 1890	Price of influenza vaccine is expensive (USD 9–15) and not competitive with international suppliers Link between seasonal influenza vaccination and pandemic preparedness noted as a challenge	Bio Farma primarily provides influenza vaccines for Hajj pilgrims through the private market Limited influenza vaccine production scale leads to the high vaccine price for the local market	NRA is at maturity level 3 ^a^ WHO has prequalified vaccines produced by Bio Farma but not its influenza vaccine ^c^
Serbia	Influenza vaccination policy in place but needs updating to align with global practices Government commitment to R&D ecosystem strengthening, including through the establishment of the BIO4 R&D complex Locally produced products receive a 5% price preference adjustment but would need to be eliminated with Serbia’s European Union accession process	Low demand for vaccines limits scale-up of local production; public awareness of influenza vaccination needs to be improved The role of the influenza vaccine manufacturer, Torlak Institute, as a stakeholder in national pandemic preparedness needs reinforcing	Torlak Institute is long-established and received NRA support to upgrade facilities to full cGMP standards, which may support export of the vaccine Training and maintaining workforce are challenges and turnover is high	NRA is at maturity level 3 ^a^
Viet Nam	Long-term strategy for influenza vaccine manufacturing, which provides demand estimates and identified target populations	Imported influenza vaccines estimated to be USD 7–10/dose; local vaccine produced by Institute of Vaccines and Medical Biologicals (IVAC) estimated to be USD 3–4/dose Seasonal influenza vaccination not considered within pandemic preparedness planning	Lack of business model to sustain production IVAC maintains animal facilities to source eggs for influenza vaccine production	NRA is at maturity level 3 ^a^

^a^ Maturity level identified by WHO/Pan American Health Organization (PAHO) lists of NRAs: https://www.who.int/publications/m/item/list-of-nras-operating-at-ml3-and-ml4 (accessed on 23 July 2025). ^b^ Brazil’s NRA is listed as an NRA of regional reference: https://www.paho.org/es/autoridades-regulatorias-referencia (accessed on 23 July 2025). ^c^ WHO PQ status confirmed by: https://extranet.who.int/prequal/vaccines/list-prequalified-vaccines (accessed on 23 July 2025).

## Data Availability

Anonymized data collected for this study will be made available by request from the authors.

## Supplementary Materials

The following supporting information can be downloaded at: https://www.mdpi.com/article/10.3390/vaccines13111160/s1, S1: Manufacturers survey, S2: Agreement of sustainability factors by domain, and S3: Differences in perceptions of importance of sustainability factors.

## Abbreviations

The following abbreviations are used in this manuscript:

CEPI	Coalition for Epidemic Preparedness Innovations
cGMP	Current Good Manufacturing Practices
GAP	Global Action Plan for Influenza Vaccines
HIC	High-income country
IP	Intellectual property
IVAC	Institute of Vaccines and Medical Biologicals
LMICs	Low- and middle-income countries
MNC	Multinational company
NH	Northern Hemisphere
NRA	National regulatory authority
PAHO	Pan American Health Organization
PQ	Prequalification
R&D	Research and development
SH	Southern Hemisphere
WHO	World Health Organization
