# Supplementary material for: Sustaining Local Production of Influenza Vaccines: A Global Study of Enabling Factors Among Vaccine Manufacturers
Source: Vaccines (Basel). 2025 Nov 14;13(11):1160. doi: 10.3390/vaccines13111160 (PMC12656779; doi:10.3390/vaccines13111160)
Supplement: Supplementary file 1 [file vaccines-13-01160-s001.zip › vaccines-3943259-supplementary.pdf]

Supplementary materials

Survey instrument

S1: Manufacturers survey (p. 2)

Supporting data

S2. Agreement of sustainability factors by domain (p. 10)

S3: Differences in perceptions of importance of sustainability factors (p. 13)

S1: Manufacturers survey

Survey focal point

The following information will be collected to identify the survey focal point. This information will only be used if we need to contact you for any follow-up questions.

1. **Contact information**
   Focal point name:
   Focal point email:
   Company/organization name:
   Country:

Manufacturer characteristics

This section aims to collection basic characteristics regarding your company.

1. **Please identify the management structure of your company.**
   - ☐ Non-profit
   - ☐ Private
   - ☐ State/government owned
   - ☐ Other – *Please specify:*

2. **What is the overall product landscape in your company?** *Please indicate the number of influenza vaccines (both seasonal and pandemic/pre-pandemic) and other vaccines (not including influenza) that are approved by a national regulatory authority (NRA) and WHO prequalified. Note that pandemic influenza vaccine would include the 2009 A/H1N1 vaccine and pre-pandemic influenza vaccine could include those for A/H2N2, A/H5N1, A/H7N9, etc. Also, note that an approved pre-pandemic influenza vaccine would include those approved through a mock dossier by a national regulatory authority.*

Product	Approved by NRA	WHO prequalified
Seasonal influenza vaccines		
Monovalent pandemic/pre-pandemic influenza vaccines		
Other vaccines (not including influenza)		

3. **Please estimate the annual installed production capacity (in total doses) for all vaccines produced by your company.**
   - ☐ <5 million doses/year
   - ☐ 5-25 million doses/year
   - ☐ 26-100 million doses/year
   - ☐ >100 million doses/year

4. **For the most recent influenza seasons, what <u>percentage</u> of your seasonal influenza vaccine production output was supplied to the public and private markets?** *If your company no longer produces an influenza vaccine or doesn't produce one for a certain hemisphere, please write "N/A."*

Influenza season	Public market	Private market
2022-23 Northern Hemisphere Season		
2022 Southern Hemisphere Season		

5. **Do other vaccine manufacturers supply seasonal influenza vaccines in your country?**
   - ☐ Yes
   - ☐ No
   - ☐ Unsure

6. **Does your company have a procurement agreement with your country's government for the influenza vaccine?** *Please note that this would apply to both seasonal influenza vaccines (annual or multi-year procurement) and pandemic influenza vaccines (advance purchase agreement). Please check all that apply.*
   - ☐ Yes, for procurement of seasonal influenza vaccines
   - ☐ Yes, for advance purchase of pandemic influenza vaccines
   - ☐ No

7. **Does your company supply a seasonal influenza vaccine to a regional or global procurement mechanism (e.g., the PAHO Revolving Fund, UNICEF)?**
   - ☐ Yes
   - ☐ No

Enabling environment for sustainable production of influenza vaccines

1. **Did demand for your seasonal influenza vaccine increase after the 2009-10 influenza A/H1N1 pandemic?** *If your company did not produce a seasonal influenza vaccine during the 2009-10 influenza A/H1N1 pandemic, please select "Not applicable".*
   - ☐ Yes *(go to question 2)*
   - ☐ No *(go to question 3)*
   - ☐ Not applicable *(go to question 3)*

2. **Was your company able to sustain that demand? If so, how?**

3. **Did demand for your seasonal influenza vaccine increase during the COVID-19 pandemic?** *If your company did not produce a seasonal influenza vaccine during the COVID-19, please select "Not applicable".*
   - ☐ Yes *(go to question 4)*
   - ☐ No *(go to question 5)*
   - ☐ Not applicable *(go to question 5)*

4. **Does your company have specific plans to sustain that increase in demand experienced during the COVID-19 pandemic?** *Please specify.*

5. **In the past five years, has your company been involved in national pandemic influenza preparedness planning efforts?** *Please check all that apply.*
   - ☐ Yes, as part of development of national pandemic preparedness plan
   - ☐ Yes, as part of pandemic influenza simulation exercise (e.g., tabletop, functional, or full-scale exercises)
   - ☐ Other – *Please specify:*
   - ☐ Not involved in national pandemic influenza preparedness planning

6. **Are you aware if your country has specific policies that support local production of vaccines?**
   - ☐ Yes *(go to question 7)*
   - ☐ No *(go to question 8)*
   - ☐ Unsure *(go to question 8)*

7. **Please specify which policies your country has in place to support local production of vaccines.**

8. **Has your country's government ever provided direct support to your company for local production of vaccines?** *Please check all that apply.*
   - ☐ Commercial capital
   - ☐ Grants
   - ☐ Loans
   - ☐ Provision of low-cost land
   - ☐ Subsidies
   - ☐ Tax and duty exemptions for imported inputs
   - ☐ Other form of direct support – *Please specify:*
   - ☐ No direct support or unsure of direct support

9. **Does your country provide indirect support for local production of vaccines?** *Please check all that apply.*
   - ☐ Development of intellectual property regimes
   - ☐ Development of investment policies
   - ☐ Development of national priority lists of medical products
   - ☐ Development of pool procurement mechanisms
   - ☐ Encouragement of regulatory harmonization
   - ☐ Facilitation of access to foreign markets
   - ☐ Facilitation of international cooperation for local production
   - ☐ Facilitation of joint ventures
   - ☐ Facilitation of relevant technology transfer
   - ☐ Improvement of the financing of health services

☐ Introduction of relevant pricing policies
☐ Regulatory system strengthening
☐ Support for incremental innovation and production
☐ Other forms of indirect support – *Please specify:*
☐ No indirect support or unsure of indirect support

10. **Please review each factor and indicate its level of importance for sustaining local production of influenza vaccines.** *A set of pre-defined sustainability factors are provided across four main areas: policy coordination and coherence; health systems and public health priorities; vaccine research, development, and manufacturing; and national regulatory authority and vaccine approval and regulation.*

Please indicate the level of importance according to the following scale:
- *1: Not at all important*
- *2: Relatively unimportant*
- *3: Neutral – neither important nor unimportant*
- *4: Moderately important*
- *5: Critically important*
- *N/A: Not applicable*

Policy coordination and coherence

**Activity/capacity/capability**	**Level of Importance**					
	**(1)**	**(2)**	**(3)**	**(4)**	**(5)**	**N/A**
Coherence among health, industrial, and economic policies that promote local production	☐	☐	☐	☐	☐	☐
Government/public sector subsidies for local production	☐	☐	☐	☐	☐	☐
Impact of multilateral and bilateral trade agreements on commercialization and import/export of products	☐	☐	☐	☐	☐	☐
International sanctions and border control issues	☐	☐	☐	☐	☐	☐
National influenza prevention and control policies, including for vaccination, that are based on local burden of disease	☐	☐	☐	☐	☐	☐
National policies to generate skilled local biomanufacturing workforce	☐	☐	☐	☐	☐	☐
Political will, stability, long-term vision, and strategic planning	☐	☐	☐	☐	☐	☐

Health systems and public health priorities

Activity/capacity/capability	Level of Importance					
	(1)	(2)	(3)	(4)	(5)	N/A
Identification of target groups for influenza vaccination	☐	☐	☐	☐	☐	☐
Influenza disease and economic burden data are known in country	☐	☐	☐	☐	☐	☐
Influenza vaccine cost-effectiveness data are known in country	☐	☐	☐	☐	☐	☐
Local manufacturer is identified as a stakeholder for pandemic influenza preparedness planning (e.g., as a participant in simulation exercises)	☐	☐	☐	☐	☐	☐
National pandemic influenza preparedness plan takes into consideration seasonal influenza vaccination	☐	☐	☐	☐	☐	☐
National pandemic influenza preparedness plan takes into consideration local vaccine manufacturer	☐	☐	☐	☐	☐	☐
Public awareness of local manufacturer/locally produced influenza vaccine	☐	☐	☐	☐	☐	☐
Public awareness of risk of seasonal and pandemic influenza	☐	☐	☐	☐	☐	☐
Regional/pooled procurement of influenza vaccines	☐	☐	☐	☐	☐	☐
Seasonal influenza vaccination included in health insurance schemes or directly provided by public sector	☐	☐	☐	☐	☐	☐
Vaccine procurement and delivery strategies and infrastructure	☐	☐	☐	☐	☐	☐

Vaccine research, development, and manufacturing

Activity/capacity	Level of Importance					
	(1)	(2)	(3)	(4)	(5)	N/A
Access to research and development networks and partnerships	☐	☐	☐	☐	☐	☐
Access to advocacy networks and partnerships, including industry associations	☐	☐	☐	☐	☐	☐
Budget and program for research and development, process development and optimization, and expansion of capacities	☐	☐	☐	☐	☐	☐
Business and strategic plans to address supply chain issues, market opportunities, and risk mitigation strategies	☐	☐	☐	☐	☐	☐

Chemistry, manufacturing, and controls, including seed development, bulk manufacturing, formulation, yields, and process validation	☐	☐	☐	☐	☐	☐
Competition with multinational manufacturers	☐	☐	☐	☐	☐	☐
Demand forecasting and marketing strategy for seasonal influenza vaccines	☐	☐	☐	☐	☐	☐
Development or sourcing of raw materials (e.g., eggs)	☐	☐	☐	☐	☐	☐
Facility design and construction	☐	☐	☐	☐	☐	☐
Maintenance of equipment	☐	☐	☐	☐	☐	☐
Number of vaccine products manufactured	☐	☐	☐	☐	☐	☐
Procurement, installation, and validation of equipment	☐	☐	☐	☐	☐	☐
Quality management, including compliance with current Good Manufacturing Practices (cGMP)	☐	☐	☐	☐	☐	☐
Reinvestment of portion of revenues in R&D	☐	☐	☐	☐	☐	☐
Strategic selection of technologies	☐	☐	☐	☐	☐	☐
Workforce availability, recruitment, retention, and training	☐	☐	☐	☐	☐	☐

National regulatory authority and vaccine approval

Activity/capacity	Level of Importance					
	(1)	(2)	(3)	(4)	(5)	N/A
Capacity to conduct clinical trials	☐	☐	☐	☐	☐	☐
Capacity to prepare and submit regulatory filings	☐	☐	☐	☐	☐	☐
Export know-how and processes	☐	☐	☐	☐	☐	☐
National regulatory authority maturity level	☐	☐	☐	☐	☐	☐
Regional/global regulatory harmonization	☐	☐	☐	☐	☐	☐
Relationship between manufacturer and national regulatory authority	☐	☐	☐	☐	☐	☐
WHO Prequalification	☐	☐	☐	☐	☐	☐

11. Please identify and rank the 10 most impactful sustainability factors for local production of influenza vaccines by your company.

Note that all previously described factors across the four main areas should be taken into consideration; all factors are listed below in alphabetical order. Of the 10 factors that are identified, please rank them 1-10, with 1 being the most impactful.

Factor	Ranking
Access to research and development networks and partnerships	
Access to advocacy networks and partnerships, including industry associations	
Budget and program for research and development, process development and optimization, and expansion of capacities	
Business and strategic plans to address supply chain issues, market opportunities, and risk mitigation strategies	
Capacity to conduct clinical trials	
Capacity to prepare and submit regulatory filings	
Chemistry, manufacturing, and controls, including seed development, bulk manufacturing, formulation, yields, and process validation	
Coherence among health, industrial, and economic policies that promote local production	
Competition with multinational manufacturers	
Demand forecasting and marketing strategy for seasonal influenza vaccines	
Development or sourcing of raw materials (e.g., eggs)	
Export know-how and processes	
Facility design and construction	
Government/public sector subsidies for local production	
Identification of target groups for influenza vaccination	
Impact of multilateral and bilateral trade agreements on commercialization and import/export of products	
Influenza disease and economic burden data are known in country	
Influenza vaccine cost-effectiveness data are known in country	
International sanctions and border control issues	
Local manufacturer is identified as a stakeholder for pandemic influenza preparedness planning (e.g., as a participant in simulation exercises)	
Maintenance of equipment	
National influenza prevention and control policies, including for vaccination, that are based on local burden of disease	
National pandemic influenza preparedness plan takes into consideration seasonal influenza vaccination	
National pandemic influenza preparedness plan takes into consideration local vaccine manufacturer	
National policies to generate skilled local biomanufacturing workforce	
National regulatory authority maturity level	
Number of vaccine products manufactured	
Political will, stability, long-term vision, and strategic planning	
Procurement, installation, and validation of equipment	
Public awareness of local manufacturer/locally produced influenza vaccine	

Public awareness of risk of seasonal and pandemic influenza	
Quality management, including compliance with current Good Manufacturing Practices (cGMP)	
Regional/global regulatory harmonization	
Regional/pooled procurement of influenza vaccines	
Reinvestment of portion of revenues in R&D	
Relationship between manufacturer and national regulatory authority	
Seasonal influenza vaccination included in health insurance schemes or directly provided by public sector	
Strategic selection of technologies	
Vaccine procurement and delivery strategies and infrastructure	
WHO Prequalification	
Workforce availability, recruitment, retention, and training	

12. **Has your company implemented specific strategies to support sustainable production of influenza vaccines across the four domains (policy coordination and coherence; health systems and public health priorities; vaccine research, development, and manufacturing; and national regulatory authority and vaccine approval)?** *If any or all of these domains do not apply to your company, please type "Not applicable".*

    - Policy coordination and coherence:
    - Health systems and public health priorities:
    - Vaccine research, development, and manufacturing:
    - National regulatory authority and vaccine approval:

13. **Are there other factors that you would consider being important for sustaining local production of influenza vaccines?**

S2. Agreement of sustainability factors by domain

Group (n)	Friedman Test p^1	Kendall's W^2	Top factor(s)
Policy coordination and coherence			
All manufacturers (12)	<.001	0.423	Influenza prevention and control policies
Private (7)	<.001	**0.605**	Influenza prevention and control policies
Public (5)	.14	0.322	Policy coherence; political will, stability, vision, strategic planning
Smaller (≤ 25M production capacity) (6)	.111	0.287	Influenza prevention and control policies
Larger (> 25M production capacity) (6)	<.001	**0.674**	Policy coherence; influenza prevention and control policies
Lower-MIC (3)	.210	0.467	Policy coherence; influenza prevention and control policies
Upper-MIC (7)	.003	0.477	Policy coherence
HIC (2)	.092	**0.907**	Influenza prevention and control policies
100% supplied (9)	<.001	**0.604**	Influenza prevention and control policies
<100% supplied (2)	.423	**0.5**	Policy coherence; subsidies for local production; influenza prevention and control policies; workforce policies; political will, stability, vision, strategic planning
Multiple markets (5)	.002	**0.682**	Influenza prevention and control policies
Single market (6)	.004	**0.535**	Policy coherence; influenza prevention and control policies
Multiple seasons (6)	.003	**0.544**	Influenza prevention and control policies
Single season (5)	.002	**0.716**	Policy coherence; influenza prevention and control policies
Exporter (4)	.013	**0.673**	Influenza prevention and control policies
Non-exporter (8)	.011	0.344	Policy coherence
Health system and public health priorities			
All manufacturers	.005	0.211	Influenza burden data
Private (7)	.092	0.233	Risk awareness of influenza
Public (5)	.034	0.391	Influenza burden data
Smaller (≤ 25M production capacity) (6)	.088	0.274	Influenza vaccination target groups
Larger (> 25M production capacity) (6)	.039	0.318	Influenza burden data; risk awareness of influenza
Lower-MIC (3)	.232	0.428	Influenza vaccination target groups; influenza burden data; influenza vaccine cost-effectiveness data
Upper-MIC (7)	.014	0.317	Influenza burden data

HIC (2)	.029	**1.0**	Influenza vaccination target groups; risk awareness of influenza; pooled procurement of influenza vaccines; seasonal influenza vaccination in health insurance scheme; vaccine procurement and delivery strategies/infrastructure
100% supplied (9)	.039	0.212	Risk awareness of influenza
<100% supplied (2)	.301	**0.588**	Influenza vaccination target groups; influenza burden data; influenza vaccine cost-effectiveness data
Multiple markets (5)	.118	0.308	Risk awareness of influenza
Single market (6)	.039	0.318	Influenza vaccination target groups; influenza burden data
Multiple seasons (6)	.008	0.395	Influenza vaccination target groups
Single season (5)	.013	0.447	Influenza burden data; influenza vaccine cost-effectiveness data; pandemic preparedness plan includes seasonal vaccination; pandemic preparedness plan considers local manufacturer
Exporter (4)	.006	**0.613**	Risk awareness of influenza; seasonal influenza vaccination in health insurance scheme; vaccine procurement and delivery strategies/infrastructure
Non-exporter (8)	.001	0.364	Influenza burden data; influenza vaccine cost-effectiveness data
Vaccine R&D and manufacturing			
All manufacturers (12)	.012	0.167	Quality management, including cGMP compliance
Private (7)	.034	0.252	Quality management, including cGMP compliance
Public (5)	.025	0.367	Equipment maintenance; quality management, including cGMP compliance
Smaller (≤ 25M production capacity) (6)	.072	0.263	R&D budget and program
Larger (> 25M production capacity) (6)	.007	0.353	Quality management, including cGMP compliance
Lower-MIC (3)	.570	0.298	CMC; demand forecasting and marketing strategy; raw materials; quality management, including cGMP compliance; strategic selection of technologies; workforce
Upper-MIC (7)	.051	0.237	Quality management, including cGMP compliance
HIC (2)	.012	**1.0**	R&D budget and program; demand forecasting and marketing strategy; reinvestment in R&D
100% supplied (9)	.010	0.227	Quality management, including cGMP compliance
<100% supplied (2)	.381	**0.534**	R&D budget and program; demand forecasting and marketing strategy; equipment maintenance; number of vaccines produced; quality

			management, including cGMP compliance; workforce
Multiple markets (5)	.039	0.346	Demand forecasting and marketing strategy
Single market (6)	.197	0.215	Quality management, including cGMP compliance; workforce
Multiple seasons (6)	.044	0.283	Demand forecasting and marketing strategy
Single season (5)	.044	0.34	Business and strategic plans; quality management, including cGMP compliance
Exporter (4)	.078	0.389	Demand forecasting and marketing strategy
Non-exporter (8)	.062	0.202	Quality management, including cGMP compliance
Vaccine approval and regulation			
All manufacturers (12)	.002	0.290	Regulatory filing capacity
Private (7)	.080	0.268	Regulatory filing capacity
Public (5)	.016	**0.519**	Regulatory filing capacity
Smaller (≤ 25M production capacity) (6)	.008	0.487	Regulatory filing capacity
Larger (> 25M production capacity) (6)	.032	0.384	Regulatory filing capacity
Lower-MIC (3)	.342	0.377	Regulatory filing capacity; NRA maturity level
Upper-MIC (7)	.004	0.448	Regulatory filing capacity
HIC (2)	.062	**1.0**	Regulatory filing capacity; export know-how and processes
100% supplied (9)	.050	0.233	Regulatory filing capacity
<100% supplied (2)	.174	**0.75**	Regulatory filing capacity; NRA maturity level; manufacturer and NRA relationship
Multiple markets (5)	.168	0.303	Regulatory filing capacity
Single market (6)	.006	**0.503**	Regulatory filing capacity; manufacturer and NRA relationship
Multiple seasons (6)	.033	0.38	Regulatory filing capacity
Single season (5)	.064	0.397	Regulatory filing capacity; NRA maturity level
Exporter (4)	.189	0.364	Regulatory filing capacity
Non-exporter (8)	.012	0.341	Regulatory filing capacity; NRA maturity level

[1] Significance set at $p < .05$ (note that significant values are highlghted in green, non-signficant dta are highlighted in red)

[2] For the Kendall's coefficient of concordance, values above 0.5 indicate a good level of agreement

S3: Differences in perceptions of importance of sustainability factors

Sustainability factor	Statistically significant differences[1] (post-hoc analysis[2])
**Coherence among health, industrial, and economic policies that promote local production**	Country income classification: p=.033 (post-hoc analyses not significant, p > .017)
Government/public sector subsidies for local production	
Impact of multilateral and bilateral trade agreements on commercialization and import/export of products	
International sanctions and border control issues	
National influenza prevention and control policies, including for vaccination, that are based on local burden of disease	
National policies to generate skilled local biomanufacturing workforce	
Political will, stability, long-term vision, and strategic planning	
**Identification of target groups for influenza vaccination**	Vaccine market: Single market suppliers rated higher than multi-market suppliers, exact p=.030
**Influenza disease and economic burden data are known in country**	Country income classification: p=.004 (Lower-MIC, p=.015, and upper-MIC, p=.004, rated higher than HIC)
**Influenza vaccine cost-effectiveness data are known in country**	Country income classification: p=.029 (post-hoc analyses not significant, p > .017)<br><br>Export status: Non-exporters rated higher than exporters, exact p=.004
**Local manufacturer is identified as a stakeholder for pandemic influenza preparedness planning**	Export status: Non-exporters rated higher than exporters, exact p=.016
**National pandemic influenza preparedness plan takes into consideration local vaccine manufacturer**	Country income classification: p=.033 (post-hoc analyses not significant, p > .017)
**National pandemic influenza preparedness plan takes into consideration seasonal influenza vaccination**	Country income classification: p=.035 (post-hoc analyses not significant, p > .017)<br><br>Export status: Non-exporters rated higher than exporters, exact p=.048
**Public awareness of local manufacturer/locally produced influenza vaccine**	Export status: Non-exporters rated higher than exporters, exact p=.048
Public awareness of risk of seasonal and pandemic influenza	
Regional/pooled procurement of influenza vaccines	
Seasonal influenza vaccination included in health insurance schemes or directly provided by public sector	
Vaccine procurement and delivery strategies and infrastructure	
**Access to advocacy networks and partnerships, including industry associations**	Country income classification: p=.05 (post-hoc analyses not significant, p > .017)
Access to research and development networks and partnerships	
**Budget and program for research and development, process development and optimization, and expansion of capacities**	Company size: Smaller manufacturers rated higher than larger, exact p=.015

**Business and strategic plans to address supply chain issues, market opportunities, and risk mitigation strategies**	Export status: Non-exporters rated higher than exporters, exact *p*=.048
Chemistry, manufacturing, and controls, including seed development, bulk manufacturing, formulation, yields, and process validation	
Competition with multinational manufacturers	
Demand forecasting and marketing strategy for seasonal influenza vaccines	
**Development or sourcing of raw materials (e.g., eggs)**	Country income classification: *p*=.033 (post-hoc analyses not significant, $p > .017$)
Facility design and construction	
**Maintenance of equipment**	Management structure: Public companies rated higher than private, exact *p*=.048
**Number of vaccine products manufactured**	Vaccine market: Single market suppliers rated higher than multi-market supplier, exact *p*=.030<br><br>Export status: Non-exporters rated higher than exporters, exact *p*=.016
Procurement, installation, and validation of equipment	
**Quality management, including compliance with current Good Manufacturing Practices (cGMP)**	Country income classification: *p*=.004 (Lower-MIC, *p*=.015, and upper-MIC, *p*=.004, rated higher than HIC)
Reinvestment of portion of revenues in R&D	
Strategic selection of technologies	
**Workforce availability, recruitment, retention, and training**	Country income classification: *p*=.029 (post-hoc analyses not significant, $p > .017$)
Capacity to conduct clinical trials	
Capacity to prepare and submit regulatory filings	
Export know-how and processes	
**National regulatory authority maturity level**	Export status: Non-exporters rated higher than exporters, exact *p*=.004
Regional/global regulatory harmonization	
**Relationship between manufacturer and national regulatory authority**	Vaccine market: Single market suppliers rated higher than multi-market suppliers, exact *p*=.030
**WHO Prequalification**	Company size: Larger manufacturers rated higher than smaller, exact *p*=.015<br><br>NH & SH vaccine formulation: Single season suppliers rated higher than multi-season suppliers, exact *p*=.030

[1] Mann-Whitney U statistically significant outputs reported for groupings with two categories or Kruskal-Wallis H statistically significant outputs reported for grouping with three or more categories; significance set at $p < .05$
[2] Post hoc pairwise analyses done following Kruskal-Wallis H tests; significance set at $p < .017$ with Bonferroni correction
